# Anisotropic Elasticity, Spin–Orbit Coupling, and Topological Properties of ZrTe_2_ and NiTe_2_: A Comparative Study for Spintronic and Nanoscale Applications

**DOI:** 10.3390/nano15020148

**Published:** 2025-01-20

**Authors:** Yasaman Fazeli, Zahra Nourbakhsh, Shahram Yalameha, Daryoosh Vashaee

**Affiliations:** 1 Chemistry & Biochemistry Department, University of Arizona, Tucson, AZ 85721, USA; yasamanfazeli@arizona.edu; 2 Faculty of Physics, University of Isfahan, Isfahan 81746-73441, Iran; yalameha93@gmail.com; 3 Department of Electrical and Computer Engineering, North Carolina State University, Raleigh, NC 27606, USA; 4 Department of Materials Science and Engineering, North Carolina State University, Raleigh, NC 27606, USA

**Keywords:** topological semimetals, ZrTe_2_ and NiTe_2_, spintronics and nanoelectronics, elastic properties, density functional theory

## Abstract

The present work investigates the interfacial and atomic layer-dependent mechanical properties, SOC-entailing phonon band structure, and comprehensive electron-topological–elastic integration of ZrTe_2_ and NiTe_2_. The anisotropy of Young’s modulus, Poisson’s ratio, and shear modulus are analyzed using density functional theory with the TB-mBJ approximation. NiTe_2_ has higher mechanical property values and greater anisotropy than ZrTe_2_. Phonon dispersion analysis with SOC effects predicts the dynamic stability of both compounds. Thus, the current research unifies electronic band structure analysis, topological characterization, and elastic property calculation to reveal how these transition metal dichalcogenides are influenced by their structural, electronic, and mechanical properties. The results obtained in this work can be used in the further development of spintronic and nanoelectronic devices.

## 1. Introduction

Transition metal ditellurides (TMTe_2_) are a subset of the broader class of transition metal dichalcogenides (TMDs). At their core, these materials manifest as a layer of transition metal atoms sandwiched between two layers of tellurium (Te) atoms, providing a foundational architecture that leads to distinctive electronic, optical, and mechanical characteristics. Much like their counterparts in the TMD family, TMTe_2_ materials have a layered constitution, individual layers of which are weakly bonded via van der Waals interactions. This layered interplay offers researchers the flexibility to exfoliate them down to monolayers, echoing the celebrated traits of graphene. Depending on the specific transition metal involved, the electronic properties of TMTe_2_ can span the spectrum from metallic to insulating. Exemplifying this diversity, compounds like MoTe_2_ lean toward semiconducting behaviors, whereas other ditellurides might show a pronounced metallic behavior. Beyond their electronic versatility, some TMTe_2_ compounds exhibit phase transitions. A case in point, MoTe_2_ can oscillate between the semiconducting hexagonal (2H) phase and its metallic orthorhombic (T’) counterpart, contingent on external factors such as temperature and pressure.

Transition metal dichalcogenides, defined by the chemical formula MX_2_, where ‘M’ encompasses metals like Zr, Ni, Ti, Mo, W, and Cr, among others, and ‘X’ signifies chalcogens, have attracted significant attention in recent years. Their potential for use in diverse applications, supported by their exemplary performance under demanding conditions, is noteworthy. Their structural composition, i.e., the X-M-X sandwich framework, facilitates a vast array of electronic behaviors, from semiconductors and semimetals to superconductors and topological phases [1,2,3]. As the wave of electronic topology research surges, the role of TMDCs, and, by extension, TMTe_2_, as carriers of topologically protected electronic states becomes even more pivotal [4,5,6].

Among the array of TMDCs, ZrTe_2_ and NiTe_2_ have attracted significant scientific interest. Characterized by their trigonal crystalline structures and alignment with the space group P-3m1 (No. 164) [7,8], these materials have a range of interesting properties waiting to be explored. NiTe_2_, for instance, is architecturally defined by a Ni atomic layer sandwiched by Te atomic layers, enabling it to adopt the unique 1T or 1H structural forms [9]. Its stability and array of applications, coupled with the recent research attention on its topological characteristics—notably, the presence of type-II Dirac points proximate to the Fermi surface—are indicative of its potential [10,11].

Zirconium-based telluride compounds have gained attention due to their varied and intriguing crystalline structures when combined with different ratios of tellurium. Notably, compounds like ZrTe, ZrTe_2_, ZrTe_3_, and ZrTe_5_ show a range of compelling physical properties. These include phenomena such as charge density waves, superconductivity [12,13], giant resistance anomalies, abnormal thermoelectric behavior [14], and Weyl phonons [15]. Recent angle-resolved photoemission spectroscopy (ARPES) studies have identified massless Dirac fermions in the ZrTe_2_ bulk phase [16]. ZrTe_2_ is predicted by DFT calculations to be a topological semimetal [17], indicating its potential for use in quantum device applications, while recent ARPES has confirmed its metallic nature [8]. The material exhibits both semimetallic and metallic properties, influenced by factors such as the temperature, external conditions, and the presence of tellurium vacancies, which can enhance its semimetallic character and lead to superconductivity [18]. At the two-dimensional limit, single-layer ZrTe_2_ shows semimetallic behavior with a low carrier density, potentially forming an excitonic insulating ground state at low temperatures [19].

**Figure 1:**
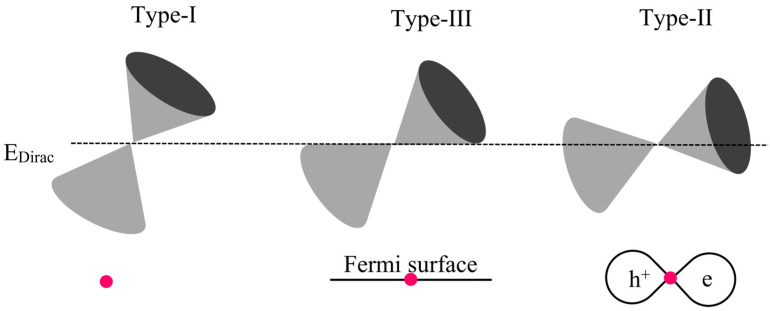
A schematic representation illustrating the various types of DSMs: type-I, type-II, and type-III. Notably, in type-II, the Fermi level (E_F_) intersects both the upper and lower Dirac cones. Adapted from reference with permission. © 2021 AIP Publishing [23].

Topological semimetals (TSMs), which encompass both Weyl semimetals (WSMs) and Dirac semimetals (DSMs) [6], are distinguished by the manner in which their energy and conduction bands intersect at, or near, the Fermi level (E_F_). These TSMs are further categorized into type-I, type-II, and type-III. In type-I, the E_F_ intersects either the upper or lower Dirac cone, whereas in type-II, it intersects both. The unique type-III category is halfway between type-I and type-II, marked by a linear Fermi surface and a flattened energy dispersion at the E_F_ (see Figure 1). DSMs exhibit Dirac points that emerge when two Weyl nodes with opposing chirality overlap at a specific k-point, making the Dirac point four-fold degenerate. Notably, DSMs differ from WSMs in that they are safeguarded by both time reversal and crystal (rotational) symmetries. Noteworthy 2D materials classified as type-II DSMs include NiTe_2_ [11], ZrTe_2_ [20], ZrTe_5_ [21], and HfTe_5_ [22]. These 2D DSMs not only display properties reminiscent of graphene but can also allow electronic device miniaturization and reduced power consumption. Additionally, they could pave the way for novel applications rooted in Weyl or Dirac fermions, such as highly fault-tolerant quantum computing, high-frequency transistors, and ultrafast optoelectronics.

ZrTe_2_ and NiTe_2_ stand out as topological materials with a diverse application spectrum. Their unique electronic band structures, augmented by strong spin–orbit coupling, endow them with pronounced spin Berry curvature. This makes them promising candidates for charge-to-spin conversion, particularly in integrated topological and ferromagnet spin–orbit torque (SOT) devices, given their noteworthy spin Hall conductivity [24,25]. A recent study [24] has demonstrated the anomalous Hall effect in ZrTe_2_ when placed near ferromagnetic materials such as Fe or Co. The magnetization of ZrO_2_ formed from the interfacial reaction between YIG (Y_3_Fe_5_O_12_, yttrium iron garnet) and ZrTe_2_ is a significant factor in the high-temperature anomalous Hall effect seen in ZrTe_2_. This can be perceived as a potential indicator of a quantum anomalous Hall effect (QAHE), especially in conjunction with topological and ferromagnetic materials. It is highly anticipated that, under these conditions, the achievement of a QAHE is viable. This revelation could pave the way for crafting efficient, magnet-free spintronic devices leveraging chiral edge modes, without the accompanying energy dissipation inherent to a QAHE system. Adding another layer of intrigue, a recent theoretical investigation [26] positioned ZrTe_2_ as a potential topological semimetal, drawing insights from the newly forged framework of topological quantum chemistry [27].

In this study, we comprehensively examine the physical properties of ZrTe_2_ and NiTe_2_ using first-principal calculations based on the Kohn–Sham density functional theory (KS-DFT). Previous theoretical, computational, and experimental research has touched upon these materials [8,16,28,29,30], addressing their structural, electronic, and optical properties. However, there is a significant information gap that limits our understanding of their full potential and applications. To bridge this gap, we have undertaken an extensive investigation, with particular attention to the electronic structures, elastic constants, and topological phases of the ZrTe_2_ and NiTe_2_ compounds. Our DFT calculations, when considering spin–orbit coupling (SOC) for ZrTe_2_ and NiTe_2_, indicate a band inversion between the Te p, Zr, and Ni d characters at the Γ point and along the Γ-A symmetry direction. Based on these data, we suggest that ZrTe_2_ and NiTe_2_ can be identified as topological semimetals.

## 2. Methodology

To analyze the structural, elastic, and topological properties of ZrTe_2_ and NiTe_2_ bulks, we employed DFT calculations using the Wien2k code (v21.2). This software addresses the Kohn–Sham equations through full potential linear augmented plane waves complemented with local orbitals. For the exchange correlation functional, we implemented the Tran–Blaha-modified Becke–Johnson exchange potential approximation (TB-mBJ) [31], factoring in the effects of SOC. For the calculation of elastic constants, we utilized the IRELAST code [32]. Additionally, the ELATOOLS code (v1.7.3) [33] was employed for the analysis of elastic constants and mechanical properties. Van der Waals (vdW) interactions were not explicitly included in these calculations. This study focuses on electronic, elastic, and topological properties, which are primarily influenced by covalent and metallic bonding. For in-plane elastic constants, such as C_11_, C_12_, and C_14_, vdW forces have minimal impact, and the results align well with values reported in the literature. While vdW interactions could affect out-of-plane constants, such as C_33_ and C_13_, their omission does not significantly alter the accuracy of the results presented here.

The phonon dispersion of ZrTe_2_ and NiTe_2_ materials was calculated using the Generalized Gradient Approximation (GGA) exchange correlation functional implemented in the Quantum ESPRESSO software (v7.0) [34], in combination with the Phonopy package (v2.9.0) [35]. The Rappe–Rabe–Kaxiras–Joannopoulos ultrasoft (rrkjus) pseudopotentials were also used. A 3 × 3 × 2 supercell was employed for the phonon band structure calculations to ensure adequate convergence of the phonon modes.

In the full potential linearized augmented plane wave (FP-LAPW) method computational approach, each unit cell is divided into muffin-tin spheres with R_MT_ radii and an interstitial region, where electrons in the muffin-tin spheres are treated separately from the electrons in interstitial region. Electrons in muffin-tin spheres, which are tightly bound and not significantly involved in chemical bonding, are typically treated using atomic-like wavefunctions within the atomic spheres. These muffin-tin sphere Kohn–Sham wave functions are expressed in spherical harmonics and are solved using a spherical potential. Kohn–Sham wave functions in interstitial region are expressed in plane waves. To guarantee the convergence of energy eigenvalues within the Kohn–Sham self-consistent framework, we set a cut-off energy threshold of 10^−5^ Ry. A separation energy of −6 Ry was established for both compounds, leading to the classification of electrons into core, valence, and quasi-core groups.

We carried out a series of tests to determine the best cut-off parameters for the calculations. For R_MT_K_max_, which represents the product of the smallest muffin-tin radius and the largest reciprocal lattice vector, we tested values ranging from 6 to 12 (a.u.)^−1^. Based on these tests, R_MT_K_max_ = 10 (a.u.)^−1^ was selected because it provided stable results, with changes in total energy falling below 10^−5^ Ry. This ensured a good balance between accuracy and computational efficiency. The magnitude of the G vector in the reciprocal lattice (G_max_) was fixed at 16.5 (Ry)^1/2^, a value that we found to be consistent with stable convergence in energy and property calculations. For k-point sampling, we used a 20 × 20 × 10 mesh in the first Brillouin zone, which was dense enough to ensure accurate descriptions of both the electronic band structures and the elastic constants. The muffin-tin radii (R_MT_) were chosen as 2.20, 2.20, and 2.35 (a.u.) for Zr, Ni, and Te, respectively, based on atomic radii and convergence requirements.

## 3. Result and Discussion

### 3.1. Structural Properties

**Figure 2:**
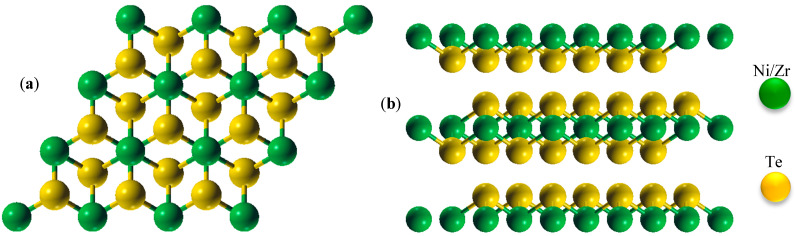
Crystal structures of ZrTe_2_ and NiTe_2_, both aligned with the P-3m1 space group, which belongs to the trigonal crystal system. (**a**) Top view: this view shows the hexagonal arrangement of Te atoms around Ni/Zr atoms, illustrating the periodic stacking in the plane of the layers. (**b**) Side view: this depicts the layered structure characteristic of the 1T phase, with alternating Te-Ni/Zr-Te layers. Green spheres represent Ni (or Zr for ZrTe_2_) atoms, and yellow spheres represent Te atoms. This figure emphasizes the trigonal prismatic coordination of Ni/Zr atoms with Te atoms.

Both ZrTe_2_ and NiTe_2_ have trigonal structures with the space group P-3m1 (No. 164). The crystal structure of these compounds is shown in Figure 2.

Table 1 presents the calculated lattice constants and the bulk modulus (B) for both compounds. The bulk modulus is a crucial parameter that characterizes the physical properties of a material. It quantifies a material’s resistance to volume change or compression, essentially gauging its rigidity. In simpler terms, a higher bulk modulus signifies that a material requires more energy to undergo deformation. From our results, it can be observed that the bulk modulus for NiTe_2_ is greater than that of ZrTe_2_, indicating that ZrTe_2_ is comparatively more compliant or softer than NiTe_2_.

When comparing the lattice parameters and c/a ratios for ZrTe_2_ and NiTe_2_ with their respective experimental values [10,16,36,37], there is a notable consistency between our computational findings and the experimental data. This suggests that the TB-mBJ approximation is aptly suited for determining structural attributes like lattice parameters.

It can be observed that the lattice parameter tends to enlarge with the increment of the atomic number of the constituting atoms. Consequently, the c/a ratio for NiTe_2_ is less than that for ZrTe_2_. The parameter c/a refers to the ratio of the out-of-plane lattice constant (c) to the in-plane lattice constant (a), providing a measure of the structural anisotropy in layered materials like ZrTe_2_ and NiTe_2_.

**Table 1:** Comparison of optimized lattice parameters (with a = b and c given in Å), cell volume (V_0_), and bulk moduli (B) for ZrTe_2_ and NiTe_2_ compounds, compared to relevant experimental data and previously reported calculations.

Compound	a [Å]	c [Å]	c/a	V_0_ [Å^3^]	B [GPa]
ZrTe_2_	3.97	7.03	1.77	86.69	16.46
Exp.	3.94 ^a^	6.62 ^a^	1.68 ^a^	89.27 ^a^	—
Others	3.90 ^a^	6.74 ^a^	1.72 ^a^	89.31 ^a^	21.90 ^b^
NiTe_2_	3.89	5.31	1.36	66.10	76.64
Exp.	3.85 ^c^	5.26 ^c^	1.37	69.03 ^e^	53.30 ^f^
Others	3.79 ^d^	5.93 ^d^	1.56	73.98 ^d^	224.6 ^d^

^a^ [16], ^b^ [8], ^c^ [10], ^d^ [38], ^e^ [39], ^f^ [40].

In our study, we found that the c/a ratio for NiTe_2_ was smaller than that for ZrTe_2_. This difference reflects the stronger in-plane bonding in NiTe_2_, which comes from the smaller atomic radius of Ni compared to Zr. We also noticed that the structural parameters for the TB-mBJ method closely matched experimental measurements. That said, it is worth mentioning that our computational values were calculated at absolute zero temperature and pressure, while the experimental data were obtained in ambient conditions.

### 3.2. Elastic Properties

Elastic constants play a pivotal role in assessing the structural and mechanical stability of materials. They are integral in numerous technological applications, as these constants are intricately linked to fundamental properties like Debye temperature, thermal expansion, and the Grüneisen parameter. Indeed, by leveraging the elastic constants of single crystals, a variety of elastic attributes can be discerned. Given that both ZrTe_2_ and NiTe_2_ have a trigonal crystal structure, they possess six distinct elastic constants: C_11_, C_33_, C_44_, C_12_, C_13_, and C_14_. To ensure mechanical stability, these constants must satisfy specific stability criteria for trigonal systems, which are detailed in Appendix A. The formulas for calculating elastic moduli, such as the Voigt and Reuss approximations, as well as the derivations for Young’s modulus and Poisson’s ratio, are provided in Appendix A. Although single-crystal elastic constants are foundational in deriving various elastic properties, the challenges of synthesizing single crystals make polycrystalline data relevant to many practical applications.

Single-crystal elastic constants (C_ij_) offer a foundation for determining various types of elastic moduli, indices, and Poisson’s ratio. Producing single-crystal specimens, however, presents challenges. These specimens are often difficult to synthesize and, in many scenarios, are impractical for large-scale use. Given these constraints, obtaining elastic constant data for polycrystalline samples becomes crucial, especially when considering real-world applications.

The elastic constants for ZrTe_2_ and NiTe_2_ compounds have been computed and are presented at zero pressure. Table 2 details these six independent elastic constants compared to prior computational findings. The differences in elastic constants between our results and previous studies can be explained by variations in computational approaches. These include choices concerning exchange correlation functionals, pseudopotentials, how van der Waals interactions are handled, convergence criteria, and other settings. Reference [8] uses CASTEP with LDA and GGA functionals. Since LDA often underestimates lattice constants, this can lead to differences in the calculated elastic constants when compared to GGA and mBj. Reference [41] employed the PAW method in VASP, which treats core and valence electrons differently and thus affected the results. Reference [42] uses PBE + SOC, which includes spin–orbit coupling and thus impacted the elastic constants. Reference [43] provides experimental values, which naturally differ from computational predictions. Reference [44] also used the CASTEP code, contributing to variations due to different computational setups and parameters.

The choice of exchange correlation functionals (LDA, GGA, or mBj) also impacts the elastic constants. LDA typically underestimates lattice constants, while GGA improves on LDA by considering density gradients, and mBj offers enhanced electronic structure predictions. Additionally, variations in k-point sampling, energy cut-offs, and convergence criteria further contribute to discrepancies, with higher k-point density and energy cut-offs generally yielding more accurate results. The GGA is particularly well suited to the calculation of elastic constants due to its ability to incorporate density gradients, providing a more accurate representation of the exchange correlation energy. Unlike the local density approximation (LDA), which often underestimates lattice constants, GGA accounts for variations in electron density, resulting in improved predictions of structural and mechanical properties. This makes GGA a reliable choice for studying materials where precise lattice parameters and elastic constants are critical.

We chose the full-potential linear augmented plane wave method with local orbitals (FP-LAPW + lo) because it provides a more precise calculation of elastic constants compared to other methods. Unlike approaches that use pseudopotentials, this method handles the full electron density and potential without approximation. It divides the unit cell into two regions: muffin-tin spheres around the atoms, where wave functions are described using spherical harmonics, and the interstitial space between atoms, where plane waves are used. This setup allows FP-LAPW to accurately capture the interactions between electrons, which is critical in calculating elastic constants. It is particularly effective for materials like ZrTe_2_ and NiTe_2_, where anisotropy plays an important role.

Of the independent single-crystal elastic constants, C_11_ and C_33_ measure the crystal’s resistance to mechanical stresses aligned with the a- (or b-) and c-crystallographic axes, respectively. As observed in Table 2 for both ZrTe_2_ and NiTe_2_ compounds, C_11_ surpasses C_33_. This implies a more densely packed structure along the c-direction compared to the a-direction. Consequently, under an applied stress, the c-axis would be more susceptible to contraction and would exhibit greater strain. The layered nature of these compounds suggests that the bonds within the ab-plane are stronger than those perpendicular to the plane [8,41,42,43,44].

Furthermore, all the elastic constants of ZrTe_2_ and NiTe_2_ compounds fulfill the conditions for mechanical stability, underscoring the inherent mechanical robustness of these TMDCs. Intriguingly, the minimal negative values observed for C_14_ in both ZrTe_2_ and NiTe_2_ do not compromise their mechanical stability, potentially hinting at minor internal strains in their optimal crystalline configurations.

The fact that C_44_ is smaller than both C_11_ and C_33_ suggests that these compounds can more easily deform under shear stress than when compressed along any individual crystallographic axis. The other elastic constants, C_12_, C_13_, and C_14_, can be regarded as off-diagonal shear components and are indicative of the structural integrity in response to diverse deformations.

Table 2 also includes findings from other computational studies. Notably, while the C_11_ values appear consistent across studies, there is notable variance in the data for other elastic constants as reported by different research groups [8,41,42,43,44]. This variance likely stems from the different computational techniques and methodologies employed by each group. The derived values for bulk modulus (B), shear modulus (G), Pugh’s ratio (B/G), Young’s modulus (E), and Poisson’s ratio (ν) are presented in Table 3. Here, B_V_ and B_R_ represent the Voigt and Reuss bulk moduli, respectively, which are averaged to obtain the bulk modulus (B).

Table 3 shows that the bulk modulus (B) estimated from the elastic constants at zero pressure aligns well with the values derived from the Birch–Murnaghan equation presented in Table 1. This correspondence underscores the reliability of our computational results, which harmonize effectively with experimental findings. The shear modulus (G) serves as a significant index of a crystal’s hardness [45]. It gauges the crystal’s resistance to plastic deformation, while the bulk modulus (B) is an indicator of its resilience against fractures [46]. The B/G ratio offers insight into the ductility or brittleness of materials. Specifically, when B/G surpasses 1.75, the material tends to be ductile. On the contrary, values below this threshold denote brittleness [45]. Our computations ascertain that at zero pressure, the B/G ratios for ZrTe_2_ and NiTe_2_ stand at 1.43 and 2.19, respectively. This implies that while ZrTe_2_ exhibits brittleness, NiTe_2_ leans towards ductility.

Additionally, Poisson’s ratio (ν) gauges a material’s responsiveness to deformation—either through expansion or contraction—when subjected to a perpendicular load. A ν value of 0.5 signifies no volumetric changes during elastic deformation. For ZrTe_2_ and NiTe_2_, the respective Poisson’s ratios are 0.22 and 0.30, both considerably lower than 0.5. This suggests that substantial volume alterations accompany their elastic deformations. When ν values hover below (or above) 0.26, the material is categorized as brittle (or ductile). Consequently, ZrTe_2_’s brittleness and NiTe_2_’s malleability corroborate the inferences made from the Pugh ratio.

Furthermore, Poisson’s ratio offers clues about the dominant interatomic forces within solids [47,48]. Values ranging between 0.25 and 0.50 hint at a prevailing central force interaction. Conversely, values outside this range point towards the dominance of non-central forces. Hence, NiTe_2_ likely has central forces taking precedence. In solids, a ν value approximating 0.33 suggests a predominantly ionic bond. In stark contrast, a value near 0.10 indicates a purely covalent bond. Given their ν values, both ZrTe_2_ and NiTe_2_ seem to favor ionic bonding. This suggests that within TMDC compounds, bonding typically comprises a blend of covalent and ionic characteristics. Poisson’s ratio also serves as a metric for the material’s shear plasticity, with higher values signifying increased ductility. Lastly, Young’s modulus stands as a pivotal parameter, encapsulating a material’s resistance to uniaxial tension.

The pronounced anisotropy in the mechanical properties, especially the shear modulus, is closely tied to the layered structure of ZrTe_2_ and NiTe_2_. This anisotropy arises from the difference between the strong in-plane bonding interactions and the weaker out-of-plane interactions in these materials. In NiTe_2_, the stronger in-plane bonding, a result of the enhanced hybridization of Ni d-states and Te p-states, contributes to the material’s higher mechanical anisotropy and greater shear modulus. In contrast, ZrTe_2_, with weaker in-plane interactions, exhibits less pronounced anisotropy.

Table 4 shows a comparison of the elastic and mechanical properties of ZrTe_2_ and NiTe_2_ in both their bulk and monolayer forms. For the bulk materials, the elastic constants (C_11_, C_12_, C_66_), Young’s modulus (E), and Poisson’s ratio (ν) were calculated in this work using the GGA functional and are expressed in GPa. For the monolayer forms, the properties were obtained from the literature and include in-plane elastic constants (C_11_, C_12_, C_66_), Young’s modulus (E), and Poisson’s ratio (ν) expressed in N/m.

The comparison highlights clear differences between the bulk and monolayer forms. For NiTe_2_, C_66_ decreases from 35.58 GPa in the bulk to 8.75 N/m in the monolayer, reflecting the greater flexibility of the monolayer. Additionally, Poisson’s ratio (ν) in NiTe_2_ increases from 0.29 in the bulk to 0.38 in the monolayer, indicating improved stretchability. Similarly, ZrTe_2_ shows significant changes in its monolayer properties, with C_66_ decreasing from 25.73 GPa in the bulk to 5.88 N/m in the monolayer. These results demonstrate how the mechanical properties of ZrTe_2_ and NiTe_2_ are influenced by dimensionality, offering important insights into their potential applications in van der Waals heterostructures, flexible electronics, and strain-engineered devices.

**Figure 3:**
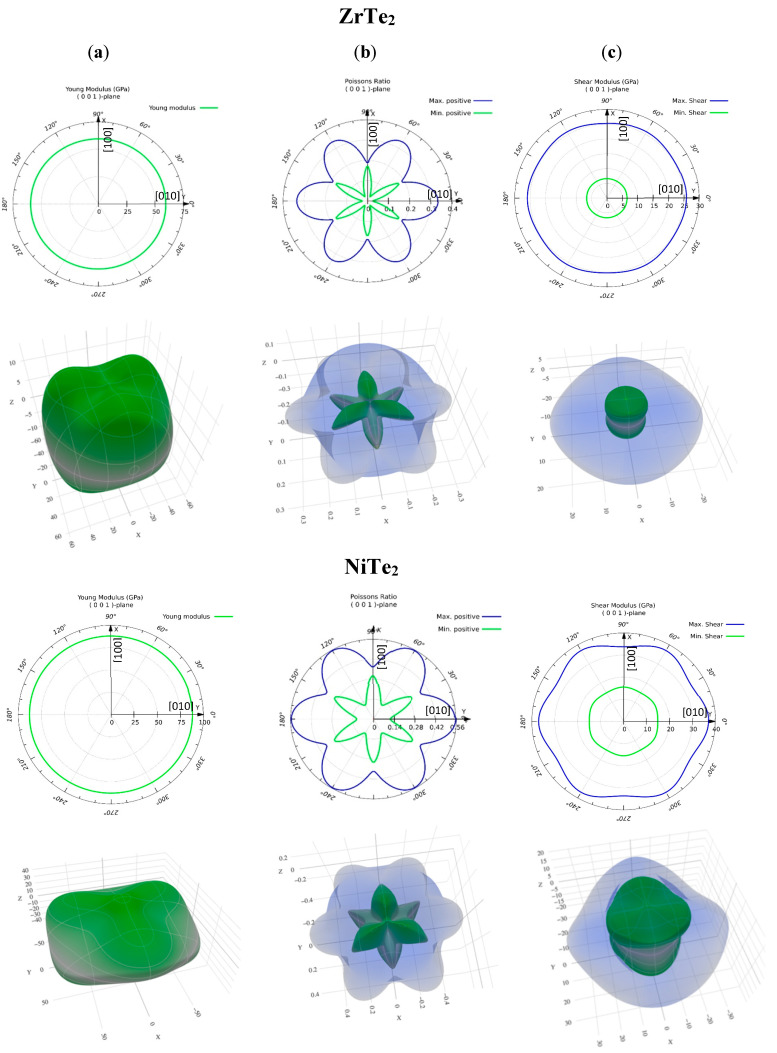
Three-dimensional visualization and corresponding 2D view of the calculated (**a**) Young’s modulus, (**b**) Poisson’s ratio, and (**c**) shear modulus for ZrTe_2_ and NiTe_2_. In (**a**), the green circular curves in the 2D view indicate isotropic elastic behavior in the (001) plane, with no directional dependence on Young’s modulus. In (**b**), the blue (green) curve represents the maximum (minimum) positive Poisson’s ratio, while the *X*-axis corresponds to the [100] direction and the *Y*-axis to the [010] direction, as labeled in the Figure. Deviations from a spherical shape in the 3D plots illustrate mechanical anisotropy, which is more pronounced in ZrTe_2_ compared to NiTe_2_. In (**c**), the blue curve represents the maximum shear modulus, while the green curve indicates the minimum shear modulus.

The anisotropic behavior of ZrTe_2_ and NiTe_2_ is effectively illustrated through the analysis of their 3D elastic property plots. For isotropic materials, the 3D representation of Young’s modulus forms a perfect sphere, with projections onto planes appearing as perfect circles. Deviations from these ideal shapes indicate anisotropy, a characteristic expected in materials with layered structures such as ZrTe_2_ and NiTe_2_. Figure 3 provides insights into the similarities and differences in the elastic properties of these materials.

Figure 3a illustrates both the 2D and 3D directional dependence of Young’s modulus for ZrTe_2_ and NiTe_2_. The green curves in the 2D polar plot exhibit a circular shape, confirming isotropic elastic behavior in the (001) plane. The constant radius of the green curves indicates that Young’s modulus has no directional dependence within the (001) plane for either material. In the 3D visualization, deviations from a spherical shape highlight the anisotropic elastic behavior of the materials. ZrTe_2_ exhibits more pronounced deviations from a spherical shape compared to NiTe_2_, indicating a higher degree of mechanical anisotropy. This is further supported by the universal anisotropy index (A^U^), which is 4.0740 for ZrTe_2_ and 1.2915 for NiTe_2_, quantitatively confirming that ZrTe_2_ is more anisotropic than NiTe_2_.

Figure 3b depicts Poisson’s ratio for each material. ZrTe_2_ exhibits moderate anisotropy, with values ranging from 0.1 to 0.4 depending on the orientation. In contrast, NiTe_2_ shows a wider range, from 0.14 to 0.56, indicating a more directionally dependent response in lateral deformation to axial stress. This suggests that NiTe_2_ undergoes more significant variation in its lateral deformation based on direction compared to ZrTe_2_. The shear modulus, as illustrated in Figure 3c, ranges from approximately 5 GPa to 30 GPa for ZrTe_2_, with notable directional dependence, while NiTe_2_ shows higher values, ranging from 10 GPa to 40 GPa, also exhibiting considerable orientation-dependent variation. The higher shear modulus values for NiTe_2_ reflect its stronger resistance to shear deformation compared to ZrTe_2_.

Both materials exhibit significant anisotropy in their mechanical properties, with variations in Young’s modulus, Poisson’s ratio, and shear modulus depending on the crystallographic orientation. While the anisotropy itself is expected due to the layered lattice structure of the 1T phase, the quantitative differences between ZrTe_2_ and NiTe_2_ provide valuable insights into their mechanical behavior. NiTe_2_ generally demonstrates higher values for all three properties, indicating stronger and more directionally dependent mechanical behavior, which could have implications for their use in specific technological applications. These findings support the understanding that deviations from spherical or circular shapes in 3D plots reflect the directional dependence of elastic properties, with NiTe_2_ showing a more pronounced directional mechanical response compared to ZrTe_2_.

The differences in the structural, electronic, and mechanical properties of ZrTe_2_ and NiTe_2_ come from their unique atomic and electronic configurations. Structurally, ZrTe_2_ has a slightly larger lattice constant than NiTe_2_, which suggests weaker interatomic bonding in ZrTe_2_. This weaker bonding contributes to its lower elastic constants and mechanical stiffness compared to NiTe_2_. Electronically, both materials behave as semimetals, but NiTe_2_ has a higher density of states near the Fermi level. This stronger electronic bonding in NiTe_2_ results in higher elastic constants and better mechanical strength. The heavier Ni atom in NiTe_2_ also enhances spin–orbit coupling effects, which further improves its mechanical stability. Mechanically, ZrTe_2_ shows greater anisotropy in its elastic properties due to its weaker in-plane bonding, making it more sensitive to directional stress. In contrast, NiTe_2_ has relatively stronger bonding both within and between layers, resulting in reduced anisotropy and a more mechanically stable response. These structural and electronic differences explain the variations in their mechanical behavior and elastic properties.

### 3.3. Electronic Band Structure and Topological Phase

The investigation of the electronic properties of compounds requires the calculation and analysis of the electron density of states and band structures. Employing the TB-mBJ approach, we have explored the band structures and the electron density of states for ZrTe_2_ and NiTe_2_. These calculations provide insights into the conductive or insulating behavior of compounds based on the electron density of states.

**Figure 4:**
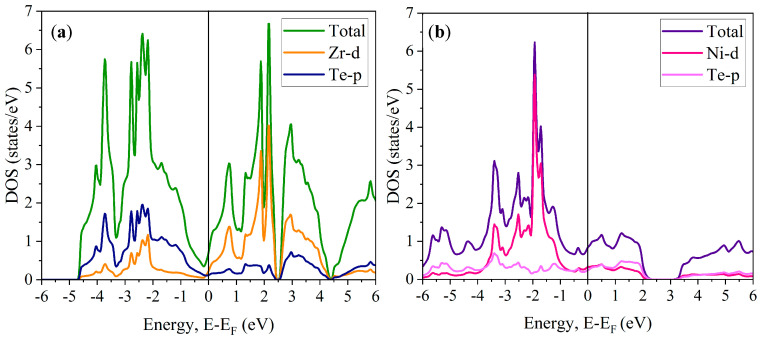
Projected electron density of states with SOC for (**a**) ZrTe_2_ and (**b**) NiTe_2_. The occupation at the Fermi level highlights the semimetallic character of these compounds.

By incorporating SOC, we have calculated the electron density of states for ZrTe_2_ and NiTe_2_, with the results presented in Figure 4. In these diagrams, the zero-energy level corresponds to the Fermi energy. The higher and lower Fermi energy levels are indicative of the conduction and valence bands, respectively. As depicted in Figure 4a for ZrTe_2_ and Figure 4b for NiTe_2_, both compounds exhibit metallic behavior. This observation is due to the Fermi level intersecting the electron density of states, thus resulting in the absence of a band gap. Furthermore, the relatively low electron density of states at the Fermi level suggests that ZrTe_2_ and NiTe_2_ are poor conductors.

The DOS at the Fermi level has a direct impact on the mechanical properties of ZrTe_2_ and NiTe_2_. A higher DOS at the Fermi level in NiTe_2_ correlates with stronger electronic interactions within the material. These interactions contribute to NiTe_2_’s greater mechanical strength, as evidenced by its higher shear modulus. In contrast, the lower DOS at the Fermi level in ZrTe_2_ indicates weaker bonding interactions, which explains its lower shear modulus and reduced mechanical resistance. This correlation suggests that the electronic structure, particularly the density of states, plays a significant role in determining the material’s mechanical stability.

Figure 4a suggests that, prior to reaching the Fermi level, the d-orbital of the Zr atom has a significant contribution, while, post the Fermi level, the p-orbital of the Te atom dominates. For NiTe_2_, the d-orbital of the Ni atom contributes predominantly below the Fermi level. Above it, the Te atom’s p-orbital slightly overshadows, with no significant contribution from the Ni atom. Our TB-mBJ + SOC calculations indicate the strong hybridization of TM (Zr, Ni) d- and Te p-like states in the near-Fermi region of the valence band of ZrTe_2_ and NiTe_2_. Similar hybridization effects have been observed in other TM-bearing tellurides, such as Cu_2_HgGeTe_4_ [51].

Bonding characteristics, particularly the hybridization of d-orbitals from the transition metals (Zr, Ni) with Te p-orbitals, play a crucial role in defining the mechanical properties of these materials. In NiTe_2_, the stronger hybridization of Ni d-states with Te p-states leads to more robust bonding within the layers. This strong bonding is reflected in NiTe_2_’s higher shear modulus, which indicates a greater resistance to shear deformation. In contrast, ZrTe_2_, with weaker Zr-Te hybridization, exhibits a lower shear modulus, demonstrating reduced mechanical strength. This comparison highlights how electronic bonding interactions directly impact the material’s mechanical behavior.

**Figure 5:**
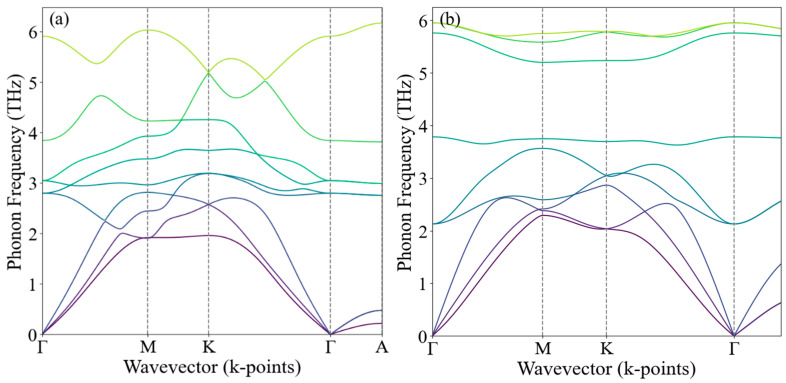
Phonon dispersion curves for (**a**) ZrTe₂ and (**b**) NiTe₂, calculated using density functional theory. The plots show the phonon frequencies as a function of the wavevector along high-symmetry directions in the Brillouin zone, following the Γ-M-K-Γ-A path. The absence of imaginary frequencies (soft phonon modes) confirms the dynamic stability of these compounds.

Figure 5 presents the phonon dispersion curves for ZrTe_2_ and NiTe_2_, calculated using density functional theory. The phonon frequencies are plotted as a function of the wavevector along high-symmetry directions in the Brillouin zone, specifically following the Γ-M-K-Γ-A path. Notably, the absence of imaginary frequencies (soft phonon modes) in both materials confirms their dynamic stability. The dispersion curves illustrate how phonon modes vary with the wavevector, providing insights into the vibrational properties of ZrTe_2_ and NiTe_2_. This analysis is crucial in understanding the thermal and mechanical behavior of these compounds, as well as their potential applications in various technological fields.

**Figure 6:**
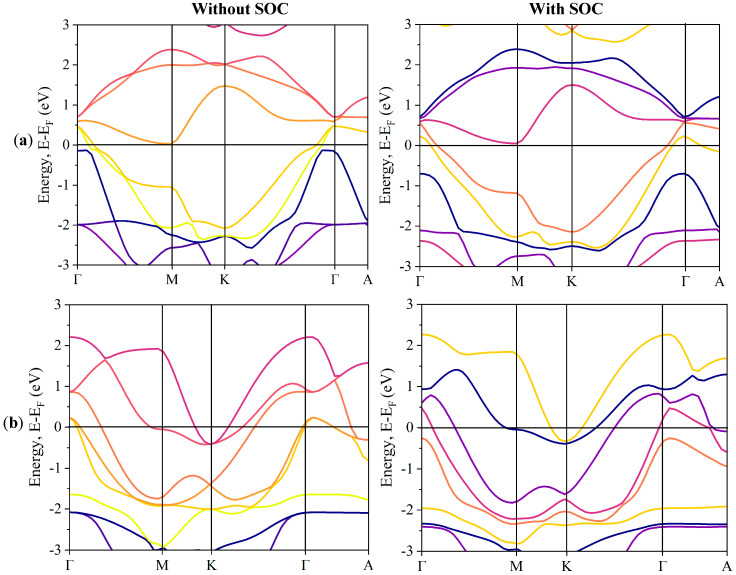
Bulk band structures of (**a**) ZrTe_2_, highlighting band inversion at the Γ point and (**b**) NiTe_2_, illustrating the intersection of valence and conduction bands at the Fermi level along the Γ-A symmetry path. The band structures are shown with and without SOC considerations for comparison.

The electronic band structure illustrates energy variations along symmetrical paths. Figure 6 shows the electronic band structures of ZrTe_2_ and NiTe_2_ along high symmetry directions (Γ-M-K-Γ-A) within the first Brillouin zone, computed using the mBJ approach with and without the inclusion of SOC at zero pressure. In this figure, the Fermi level is depicted as a horizontal line set at 0 eV. From our analysis, as shown in Figure 6a, ZrTe_2_ experiences a band inversion between the Zr d-states and Te p-states at the Γ point. For ZrTe_2_, the Zr d-state approaches the valence band, even making contact at the Γ point. In the presence of SOC, this band inversion in ZrTe_2_ becomes more pronounced: the Te p-state advances to the conduction band, and the Zr d-state ascends further within the valence band.

In contrast, the electronic band structure of NiTe_2_, as influenced by SOC and shown in Figure 6b, demonstrates band inversion at the Fermi level along the Γ-A symmetry direction, a hallmark of its topological nature. The topological properties of NiTe_2_ and ZrTe_2_ are well documented as type-II Dirac semimetals. In this study, we avoided redundant analyses, and we refer readers to previous research for detailed information [20,52,53,54,55,56,57].

The topological characteristics of NiTe_2_, particularly the band inversion near the Fermi level, have a direct influence on its mechanical stability. The presence of this band inversion along the Γ-A symmetry direction strengthens the material’s resistance to deformation, as reflected in its higher shear modulus. This topological feature enhances the bonding interactions within the material, contributing to NiTe_2_’s superior mechanical stability compared to ZrTe_2_. The electronic band structure thus plays a dual role, not only defining the material’s electronic properties but also impacting its mechanical robustness, which is vital in applications that demand both mechanical durability and electronic functionality.

To evaluate the thermodynamic stability of the structures, we calculated the cohesive energy (E_C_). This represents the amount of energy required to decompose the solid into its constituent atoms in their stable states. The cohesive energy is determined using the following equation [58]:

(1) $$E_{C}=\frac{E_{Bulk}^{Tot}-{N_{Te}E}_{Te}^{Tot}-N_{Zr/Ni}E_{Zr/Ni}^{Tot}}{N_{Te}+N_{Zr/Ni}},$$

In this equation, $E_{Bulk}^{Tot}$ represents the total energy of the bulk material, while $E_{Te}^{Tot},E_{Zr/Ni}^{Tot}$ are the total energies of each individual element. Additionally, $N_{Te},N_{Zr/Ni}$ denote the number of atoms of each element within the unit cell. The cohesive energies calculated for the ZrTe_2_ and NiTe_2_ compounds are −4.69 and −3.47 eV/atom, respectively. The negative values of these cohesive energies indicate that these structures are thermodynamically stable.

The electronic and structural properties of ZrTe_2_ and NiTe_2_ demonstrate their potential for use in practical applications in spintronic and nano-electronic devices. NiTe_2_, with strong spin–orbit coupling and a higher density of states near the Fermi level, is well suited to spintronic technologies. These properties enhance spin polarization and enable efficient charge–spin conversion, making NiTe_2_ an excellent candidate for components such as spin filters and spin transistors. ZrTe_2_ exhibits semimetallic behavior and anisotropic mechanical properties, making it ideal for nano-electronic applications that require directional conductivity or flexibility. The layered structure and mechanical robustness of ZrTe_2_ make it particularly suitable for flexible and wearable electronic devices, including bendable systems. The unique combination of electronic precision, mechanical stability, and topological robustness positions ZrTe_2_ and NiTe_2_ as promising materials for next-generation device applications.

## 4. Conclusions

Our comprehensive investigation of ZrTe_2_ and NiTe_2_ using density functional theory calculations yielded several important insights into their electronic, topological, elastic, and vibrational properties. Our study led to several novel contributions:

- We revealed significant anisotropy in the orientation-dependent mechanical properties of both compounds, with NiTe_2_ exhibiting more pronounced variations and generally higher values for Young’s modulus, Poisson’s ratio, and shear modulus compared to ZrTe_2_.
- Using the TB-mBJ method, we confirmed the type-II Dirac semimetal nature of both materials, observing distinct band inversion characteristics: ZrTe_2_ showed inversion between Zr d and Te p states at the Γ point, while NiTe_2_ exhibited band inversion along the Γ-A symmetry direction near the Fermi energy.
- Our analysis demonstrated the crucial role of spin–orbit coupling in enhancing the topological features of these materials, particularly in accentuating the band inversions.
- Phonon dispersion calculations confirmed the dynamic stability of both ZrTe_2_ and NiTe_2_, an essential consideration in their potential applications.
- By comparing ZrTe_2_ and NiTe_2_ side by side, we highlighted key differences in their mechanical and electronic properties, providing valuable insights for use in material selection in various applications.

These findings have significant implications for the field of spintronics and next-generation electronic devices. The unique combination of a layered structure, non-trivial band topology, and anisotropic mechanical properties in ZrTe_2_ and NiTe_2_ opens up new possibilities for tailored material design in advanced applications.

The mechanical properties of ZrTe_2_ and NiTe_2_, particularly their anisotropy and shear modulus, are strongly influenced by their electronic structures. The higher DOS at the Fermi level in NiTe_2_, along with its stronger d-p hybridization and topological band inversion, result in greater mechanical strength and resistance to deformation. In contrast, the weaker bonding interactions in ZrTe_2_, as indicated by its lower DOS and weaker hybridization, lead to reduced mechanical stability. These findings demonstrate that the electronic structure, particularly the DOS and bonding interactions, plays a crucial role in determining the mechanical behavior of these materials. This correlation has important implications for optimizing the mechanical and electronic performance of ZrTe_2_ and NiTe_2_ in electronic and spintronic devices.

Our work highlights the importance of considering multiple factors—including orientation-dependent mechanical properties, electronic structures, and vibrational dynamics—when evaluating these materials for practical applications. This holistic approach provides a more comprehensive understanding of ZrTe_2_ and NiTe_2_, paving the way for their optimized use in future technological innovations. Future research directions could include the experimental validation of the predicted anisotropic properties, the exploration of heterostructures involving these materials, and the investigation of their behavior under various external stimuli, such as strain or electric fields.

## Figures and Tables

**Table 2:** Comparison of elastic constants (C_ij_ in GPa) for ZrTe_2_ and NiTe_2_ compounds with previous computational findings.

Compound	C_11_ [GPa]	C_12_ [GPa]	C_13_ [GPa]	C_14_ [GPa]	C_33_ [GPa]	C_44_ [GPa]	Ref.
ZrTe_2_	68.00	13.60	6.30	−1.30	31.30	8.40	This work (mBj)
	62.397	10.95	2.613	−1.779	11.742	6.735	This work (GGA)
	67.65	12.95	8.07	1.07	32.30	6.94	[8]
	69.00	—	—	—	26.00	31.00	[41]
NiTe_2_	121.40	39.27	42.82	−7.50	72.20	16.92	This work (mBj)
	112.735	41.583	26.675	−5.498	50.948	16.391	This work (GGA)
	113.7	36.60	27.20	−6.50	45.70	11.20	[42]
	109.50	41.90	—	−10.70	52.60	20.40	[43]
	147.60	50.80	44.10	7.91	83.90	17.58	[44]

**Table 3:** Calculated elastic moduli (in GPa) for ZrTe_2_ and NiTe_2_ compounds, including Voigt bulk modulus (B_V_), Reuss bulk modulus (B_R_), bulk modulus (B), shear modulus (G), Young’s modulus (E), Pugh’s ratio (B/G), and Poisson’s ratio (ν), compared with previously reported computational results.

Compound	B_V_ [GPa]	B_R_ [GPa]	B [GPa]	G_V_ [GPa]	G_R_ [GPa]	G [GPa]	E [GPa]	B/G	ν	Ref.
ZrTe_2_	24.41	20.79	22.60	18.21	13.45	15.83	38.50	1.43	0.22	This work (mBj)
	18.76	9.81	14.29	15.86	9.72	12.79	29.55	1.12	0.16	This work (GGA)
	25.09	21.90	23.49	17.47	11.78	14.62	36.34	1.60	0.24	[8]
NiTe_2_	62.76	59.22	60.99	27.65	27.98	27.81	56.37	2.19	0.30	This work (mBj)
	51.81	43.07	47.44	25.77	21.16	23.47	60.43	2.02	0.29	This work (GGA)
	—	—	70.12	—	—	28.75	5.095	2.439	0.3196	[44]

**Table 4:** Elastic constants (C_11_, C_12_, C_66_), Young’s modulus (E), and Poisson’s ratio (ν) for ZrTe_2_ and NiTe_2_ compounds in bulk (calculated in this work using GGA) and monolayer forms (obtained from the literature).

Compound	Type	C_11_	C_12_	C_66_	E	ν	Reference
ZrTe_2_	Bulk [GPa]	62.40	10.95	25.73	29.55	0.16	This work (GGA)
	Monolayer [N/m]	28.00	16.25	5.88	—	—	[49]
NiTe_2_	Bulk [GPa]	112.73	41.58	35.58	60.44	0.29	This work (GGA)
	Monolayer [N/m]	28.08	10.58	8.75	24.09	0.38	[50]

## Data Availability

Data is contained within the article.

## Appendix A

According to reference [59], these stability criteria for a trigonal crystal system are as follows:

(A1) $$C_{11}-C_{12}>0\;\left(C_{11}+C_{12}\right)C_{33}-2C_{13}^{2}>0\;\left(C_{11}-C_{12}\right)C_{44}-2C_{14}^{2}>0.$$

From the aforementioned independent elastic constants, we can derive a theoretical elastic modulus. Two established methods for this calculation are the Voigt and Reuss approximations. Specifically for trigonal crystals, the bulk modulus in the Voigt (B_V_) and Reuss (B_R_) approaches are represented as follows [60]:

(A2) $$B_{V}=\frac{1}{9}\left(2\left(C_{11}+C_{12}\right)+C_{33}+4C_{13}\right),$$

(A3) $$B_{R}=\frac{(C_{11}+C_{12})C_{33}-2C_{13}^{2}}{C_{11}+C_{12}+2C_{33}-4C_{13}}.$$

Furthermore, the shear modulus values, G_V_ and G_R_, derived using the Voigt and Reuss methods for trigonal crystals, are formulated as follows [61]:

(A4) $$G_{V}(trigonal)=\frac{1}{30}(7C_{11}-5C_{12}+2C_{33}-4C_{13}+12C_{44}),$$

(A5) $$G_{R}\left(trigonal\right)=\frac{15}{2}{\left[\frac{2C_{11}+2C_{12}+4C_{13}+C_{33}}{C_{33}\left(C_{11}+C_{12}\right)-2C_{13}^{2}}+\frac{3C_{11}-3C_{12}+6C_{44}}{C_{44}\left(C_{11}-C_{12}\right)-C_{14}^{2}}\right]}^{-1}.$$

We can also calculate the shear modulus (G) and bulk modulus (B) as follows [62]:

(A6) $$G=\frac{1}{2}(G_{R}+G_{V}),$$

(A7) $$B=\frac{1}{2}(B_{R}+B_{V}).$$

Young’s modulus (E) and Poisson’s ratio (ν) for these elastic constants are then calculated using the following equations [63]:

(A8) $$E=\frac{9BG}{3B+G},$$

(A9) $$\nu =\frac{3B-2G}{6B+2G}.$$
